# Effect of electromigration on microstructure and properties of CeO_2_ nanopartical-reinforced Sn58Bi/Cu solder joints

**DOI:** 10.1038/s41598-024-66681-y

**Published:** 2024-07-08

**Authors:** Weiming Chen, Keke Zhang, Yuchun Fan, Chao Zhang, Nannan Wang

**Affiliations:** 1 School of Materials Science and Engineering, Henan University of Science and Technology, Luoyang, 471000 China; 2 Key Laboratory of Nonferrous Metals Science and Processing Technology in Henan Province, Luoyang, 471000 China; 3 School of Mechanical and Electrical Engineering, Luoyang Polytechnic, Luoyang, 471000 China; 4 Luoyang College, Civil Aviation Flight University of China, Luoyang, 471000 China

**Keywords:** Sn58Bi solder, CeO_2_ nanoparticle, Electromigration, Solder joint, Mechanical property, Engineering, Materials science

## Abstract

To mitigate the decrease in mechanical performance of Sn58Bi/Cu solder joints resulting from electromigration-induced damage. The CeO_2_ nanoparticles were incorporated into Sn58Bi solder by a melt-casting method, and their effects on the microstructure and properties of Sn58Bi/Cu solder joints under electromigration were investigated. The study results demonstrate that the addition of 0.125 ~ 0.5 wt% CeO_2_ nanoparticles refines the eutectic microstructure of Sn58Bi solder alloy. At an addition amount of 0.5 wt%, the composite solder alloy exhibits the maximum tensile strength of 68.9 MPa, which is 37% higher than that of the base solder. CeO_2_ nanoparticle-reinforced Sn58Bi solder can achieve excellent solderbility with Cu substrates and the joints can significantly inhibit the growth of the anodic Bi-rich layer, which is responsible for electromigration. With the extension of current stressing time, Bi-rich and Sn-rich layer are respectively formed on the anode and cathode in the joints. The intermetallic compound (IMC) layer grows asymmetrically, transitioning from a fan-shaped morphology to a flattened structure at the anode and to a thickened mountain-like morphology at the cathode. Adding the CeO_2_ nanoparticles helps to mitigate the decrease in mechanical performance caused by electromigration damage during current application to some extent. Over the current stressing period of 288 ~ 480 h, the fracture position shifts from the anodic IMC/Bi-rich interface to the cathodic Sn-rich/IMC interface. The fracture mechanism transitions from a brittle fracture characterized by plate-like cleavage to a ductile–brittle mixed fracture with fine dimples and cleavage.

## Introduction

Sn58Bi solder is widely used in micro-connections of electronic components due to its low melting point (138 °C), good wettability, and low cost^1^. However, the high content of Bi in Sn58Bi solder leads to coarse microstructure and high brittleness after soldering^2^. The Bi phase in Sn58Bi solder tends to coarsen under prolonged exposure to high temperature and high current conditions, resulting in a sharp decrease in the mechanical properties of solder joints and a weakening of their reliability, which has become a prominent issue affecting the reliability of microelectronic connections^3–7^. Therefore, to meet the demand for high reliability of solder joints in high-density microelectronic connection technology^8^, it is particularly important to improve the electromigration resistance of Sn58Bi solder joints.

Guo et al.^9^ studied the diffusion behavior of Sn atoms in Sn58Bi/Cu solder joints under current stress and found a direct correlation between the evolution of joint microstructure and temperature distribution. They observed that metal atoms in the horizontal direction are simultaneously influenced by electron wind force and thermal gradient, causing Bi atoms to migrate to the lower temperature side, while Sn atoms migrate in the opposite direction. Hadian et al.^10^ investigated the relationship between electrical resistance and microstructure of Sn58Bi/Cu solder joints under current stress, and found that the change in electrical resistance under current stress is linearly related to the accumulation of Bi at the anode, and electromigration is the main driving force for Bi diffusion in Sn.

Nanoparticle reinforcement is one of the commonly used methods for modifying lead-free solder materials^11^. Ismathullakhan et al.^12^ added 2 wt% Ag nanoparticles to Sn58Bi solder and found that Ag nanoparticles refine the β-Sn microstructure. Compared with pure Sn58Bi solder alloy, Sn58Bi solder doped with Ag nanoparticles restricts the migration of Bi atoms, leading to a 10 ~ 15% increase in shear strength of solder joints. Liu et al.^13^ reported that the addition of graphene nanoplates (GNS) also enhances the electromigration resistance of Sn58Bi/Cu solder joints. The addition of GNSs to Sn58Bi solder exhibits two competing mechanisms for electromigration behavior: one is the refinement of the microstructure, which provides more diffusion channels for Bi atoms; the other is the blocking effect on diffusing atoms. Yang et al.^14^ added 0.5 wt% Al_2_O_3_ particles to Sn58Bi solder paste and found that the addition of Al_2_O_3_ particles reduces the growth rate of IMC layer in Sn58Bi/Cu solder joints, resulting in a slight increase in the shear strength of solder joints. Kim et al.^15^ studied the effect of adding Ag-MWCNTs on Sn58Bi/Cu solder joints and found that Ag-MWCNTs act as diffusion barriers under current stress, inhibiting the migration of Sn and Cu atoms, significantly improving the electromigration reliability of Sn58Bi/Cu solder joints.

CeO_2_ nanoparticles, due to their excellent stability in high-temperature and high-pressure environments, as well as outstanding ionic and electronic conductivity, have been widely used in the fields of energy and chemical engineering^16,17^. Li et al.^18^ mixed CeO_2_ nanoparticles with Sn57Bi1Ag solder paste to successfully prepare SBA-*x*CeO_2_ composite solder with different CeO_2_ nanoparticle contents. The research results showed that CeO_2_ nanoparticles act as heterogeneous nucleation sites in the solder, refining microstructure of the solder alloy, suppressing the growth of interface IMC layer grains, and improving the shear performance of joints. The fracture mode transitions from pure brittle fracture to a mixed mode of ductile and brittleness. Therefore, in this study, CeO_2_ nanoparticle-reinforced Sn58Bi composite solder was prepared by the melt-casting method to observe and analyze the influence of CeO_2_ nanoparticles on the microstructure and mechanical properties of solder joints under different current conditions, providing theoretical and experimental basis for improving the reliability of Sn58Bi solder joints.

## Experimental materials and methods

### Experimental materials

**Figure 1:**
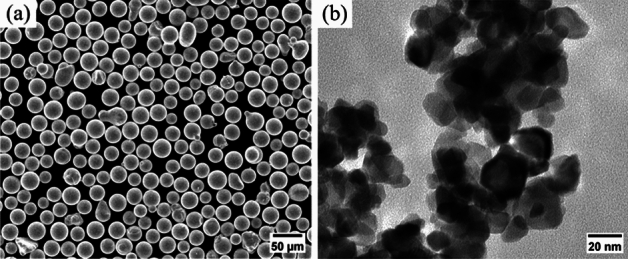
Morphological images of the raw materials used in the experiment: (**a**) SEM image of Sn58Bi particle solder alloy and; (**b**) TEM image of CeO_2_ nanoparticles.

The morphologies of the raw materials used in the experiment are shown in Fig. 1. The solder matrix used for experiment was Sn58Bi solder alloy powder (Changsha Tianjiu Metal Materials Co., Ltd., China) with a particle size of 300 mesh and a spherical shape (Fig. 1a). The CeO_2_ nanoparticles (Xuzhou Jiechuang New Materials Technology Co., Ltd., China) with a purity of 99.5%, exhibiting a granular shape with a particle size of 20 nm (Fig. 1b). The copper plate used for the soldering was T2 copper plate (Wuxi Taibang Special Steel Co., Ltd, Wuxi, China) with a purity of 99.9% and the flux used in the experiment was NC-559-V2-TF clean-free flux (AMTECH MANUFACTURING, LCC, Glastonbury, USA).

### Preparation of composite solder

**Figure 2:**
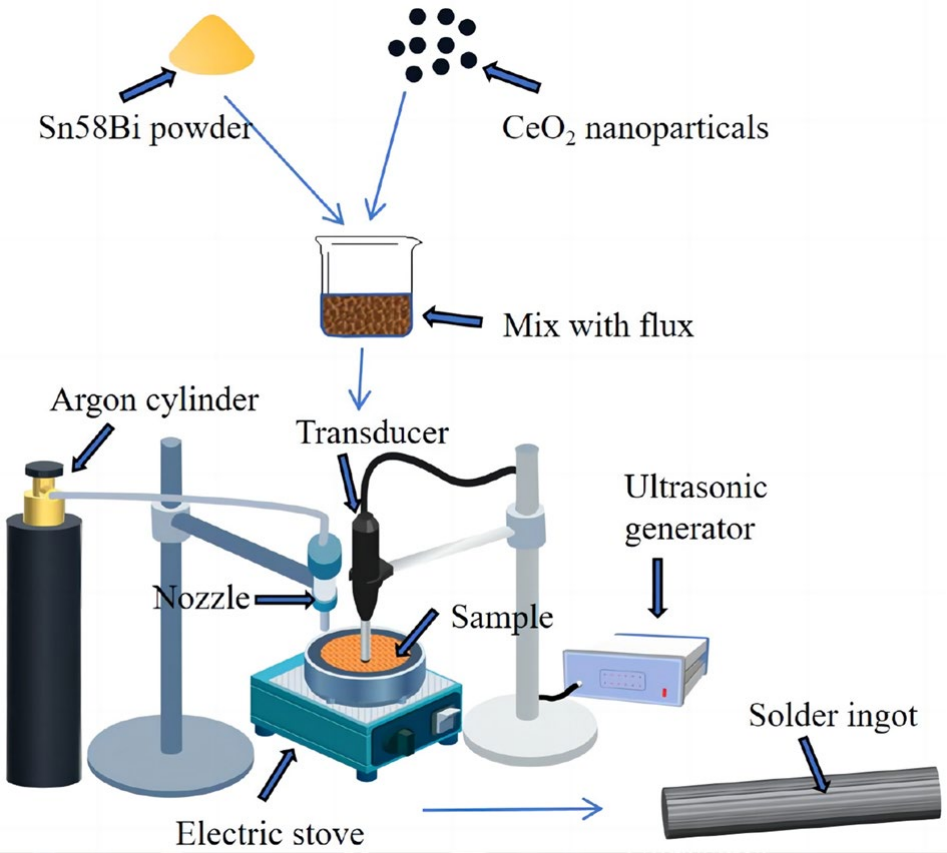
Schematic diagram of composite solder alloy preparation process.

The process of preparing the composite solder alloy is illustrated in Fig. 2. Sn58Bi solder powder and CeO_2_ nanoparticles with different mass fractions (0%, 0.125%, 0.25%, 0.5%, 1.0%, and 1.5%) were thoroughly mixed by mechanical stirring. The mixture was then mixed with flux at a ratio of 9:1 and placed in a crucible. The crucible was stirred and heated in a furnace at 200 °C. Ultrasonic treatment was applied to the liquid alloy, with a frequency of 20 kHz and a duration of 1 min, while maintaining continuous flow of argon gas to prevent oxidation. The molten alloy was poured into graphite molds and allowed to air-cool, resulting in Sn58Bi-*x*CeO_2_ composite solder alloy ingots with dimensions of 10 mm × 8 mm × 90 mm. These ingots were then mechanically processed into solder foil with a thickness of 0.1 mm for soldering experiments.

### Preparation of solder joints

**Figure 3:**
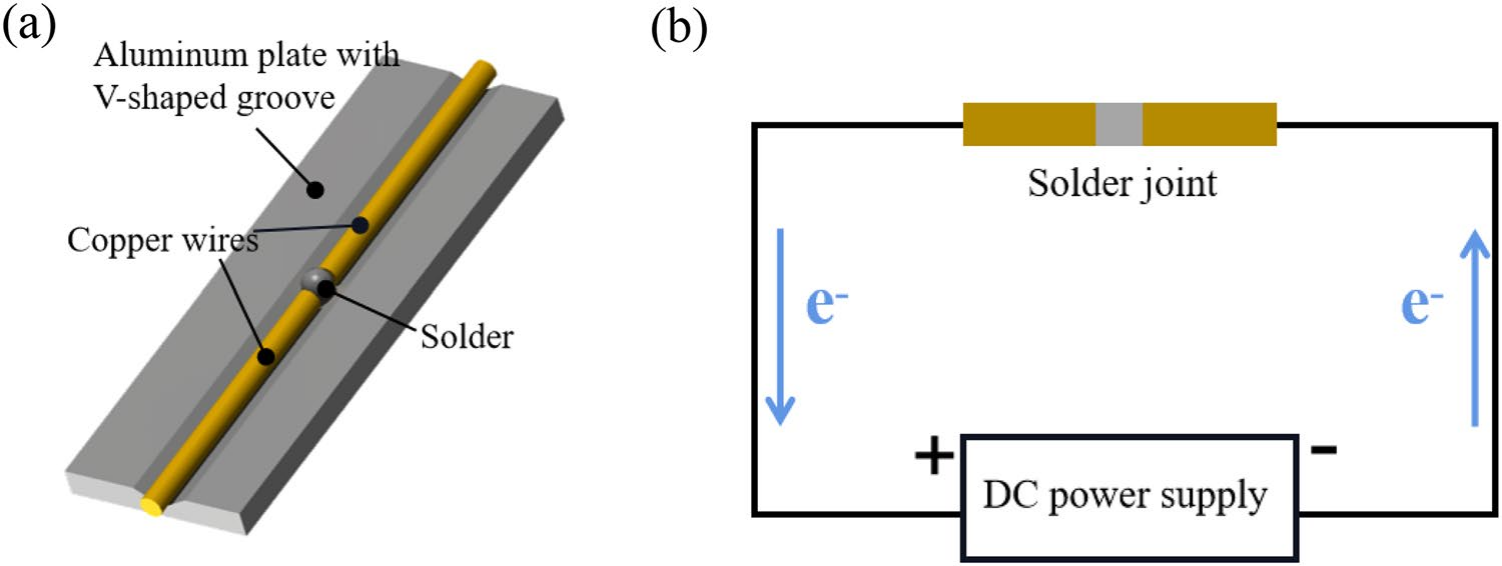
Schematic diagram of solder joint preparation and electromigration test (**a**) solder joint preparation using V-Groove; (**b**) electromigration test.

The solder joint preparation with a V-groove is illustrated in Fig. 3a. The soldering material is placed between the copper wires with a diameter of 0.8 mm, and a small amount of flux is evenly applied. The assembly is then placed in an aluminum alloy V-groove fixture and pressed firmly. The entire assembly is placed on a heating platform for soldering. The soldering temperature is set at 180 °C, and the soldering time is 120 s. After soldering, excess solder is removed by grinding.

### Electromigration test

The schematic diagram of the electromigration test is shown in Fig. 3b. Ten joints are connected in series and fixed on a copper fixture. The ambient temperature is maintained at 25 °C, and the current density is set to 7.5 × 10^3^ A/cm^2^. After 30 min of current stress, the joint stabilizes at 85 ± 2 °C, and the duration of current stress ranges from 0 to 600 h.

### Properties test

The microstructure morphology of the solder alloy and solder joints was observed using an IT-800 scanning electron microscope (SEM), and the composition of the joints was detected using an INCACH5 energy dispersive spectrometer (EDS). The melting point of the composite solder alloy was tested using a differential scanning calorimetry (DSC) melting point tester^19^. The electrical conductivity of the composite solder alloy was measured using a portable Sigma2008B1 digital conductivity meter. According to the GB/T11364-2008 standard^20^, 0.2 g of composite solder alloy was taken, covered with flux, and placed in the center of a copper substrate. The assembly was kept warm at 180 °C for 120 s. The average thickness of the interface IMC and the Bi-rich layer was measured and calculated using Image J image processing software and the equal-area method.

**Figure 4:**
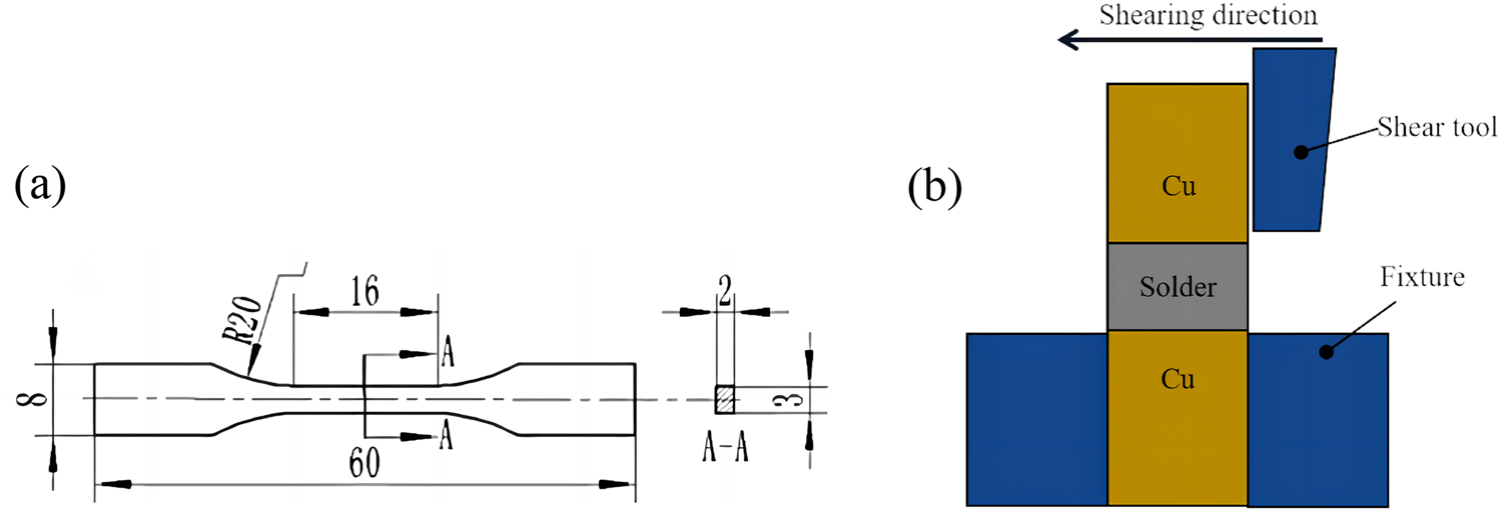
Schematic diagram of tensile test and shearing test (**a**) solder alloy tensile specimen; (**b**) solder joint shearing test.

The schematic diagram of the solder alloy tensile specimen is shown in Fig. 4a. The tensile strength of the composite solder alloy was measured using a UTM2503 electronic universal testing machine at a tensile rate of 1 mm/min. The schematic diagram of the solder joint shear test is shown in Fig. 4b. The shear force of the joint was tested using an MFM-1200 wire bonding force tester at a shear speed of 1 mm/min. Performance values were averaged over 5 measurements.

## Results and discussion

### Microstructure of composite solder alloy

**Figure 5:**
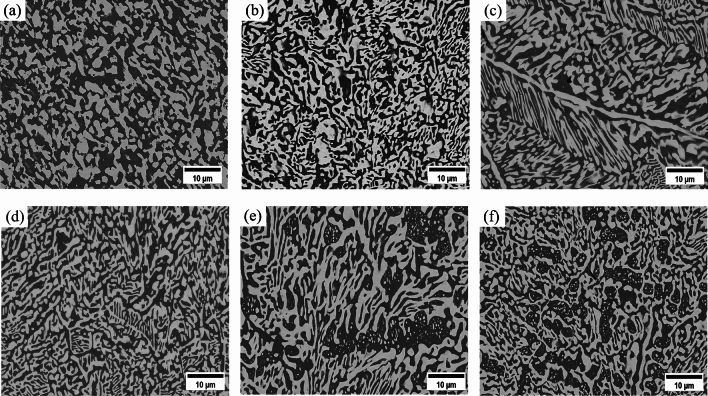
SEM backscattered electron image of microstructure of Sn58Bi-*x*CeO_2_(x = 0–1.5 wt%) composite solder: (**a**) 0 wt%; (**b**) 0.125 wt%; (**c**) 0.25 wt%; (**d**) 0.5 wt%; (**e**) 1.0 wt%; (**f**) 1.5 wt%.

The SEM backscattered electron images of the microstructure of Sn58Bi-*x*CeO_2_ composite solder alloy are shown in Fig. 5. From Fig. 5, it can be seen that the white regions represent the Bi phase, while the dark regions represent the β-Sn phase. The Sn58Bi solder consists of eutectic structure, primary β-Sn phase, and a small amount of secondary Bi particles (Fig. 5a). The eutectic structure comprises a layered structure composed of blocky and strip-like Bi phases and β-Sn phases. With the increase in the addition of CeO_2_ nanoparticles, the eutectic structure of Sn58Bi-*x*CeO_2_ composite solder becomes refined (Fig. 5b,c). When the addition amount of CeO_2_ nanoparticles reaches 0.5wt.%, the size of the Bi phase decreases from blocky to fine strip-like (Fig. 5d), and the eutectic structure changes from a layered structure to a fine mesh structure. When the addition amount exceeds 0.5wt.%, the β-Sn phase coarsens into relatively large blocks (Fig. 5e,f).

### Properties of composite solder alloy

**Figure 6:**
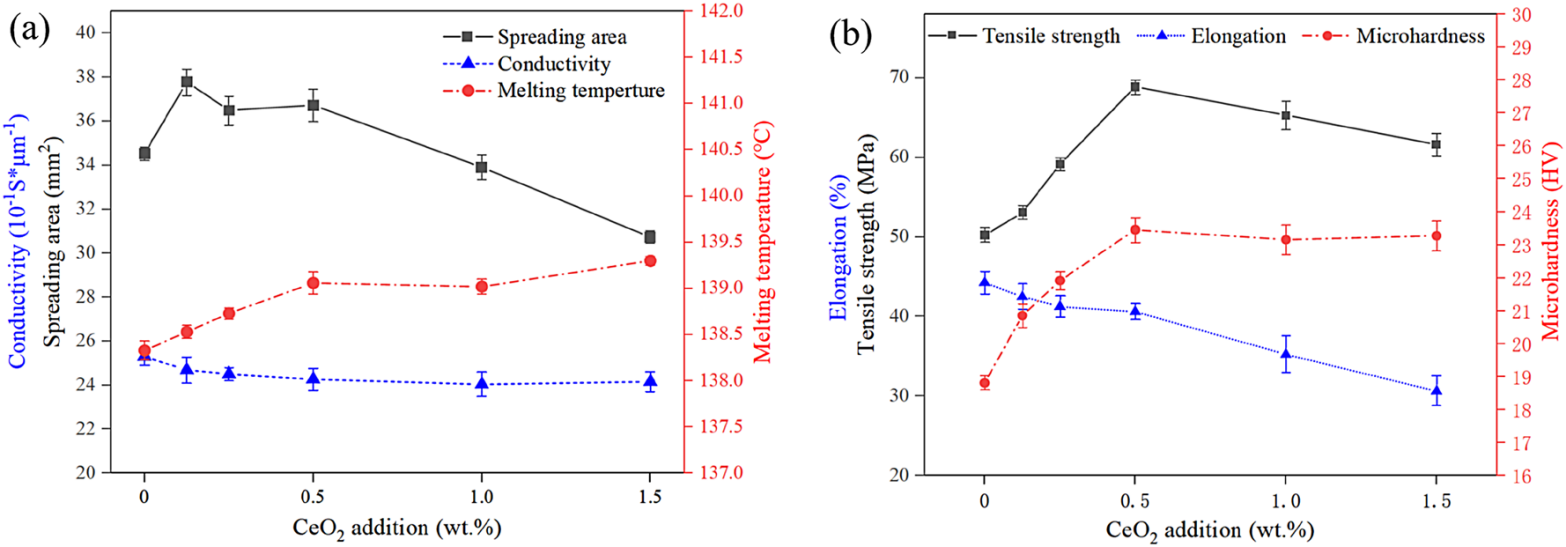
The properties of Sn58Bi-*x*CeO_2_ (x = 0 ~ 1.5 wt%)composite solder alloys (**a**) conductivity, spreading area and melting temperature; (**b**) tensile strength, microhardness, elongation.

The properties of the Sn58Bi-*x*CeO_2_ composite solder alloy are shown in Fig. 6. From Fig. 6a, it can be observed that the addition of a suitable amount of CeO_2_ nanoparticles has minimal impact on the electrical conductivity and melting point of the composite solder alloy, while it improves the wettability to some extent. When the addition amount of CeO_2_ nanoparticles ranges from 0 wt% to 1.5 wt%, the electrical conductivity of the composite solder alloy fluctuates between 2.34 S/um and 2.53 S/um, and the melting point increases only slightly from 138.7 °C for Sn58Bi to 139.1 °C for Sn58Bi-1.5CeO_2_, which still suits existing soldering processes and meets the requirements for solder alloy conductivity in microelectronic connections. When the addition amount of CeO_2_ nanoparticles ranges from 0.125 wt% to 0.5 wt%, the spreading area of the composite solder is greater than that of the base solder, with the maximum spreading area observed at 0.125 wt%, representing a 9.4% increase compared to the base solder.

The addition of CeO_2_ nanoparticles improves the tensile strength and microhardness of the Sn58Bi-*x*CeO_2_ composite solder alloy, but it leads to a certain decrease in elongation (Fig. 6b). When the addition amount of CeO_2_ nanoparticles is 0.5 wt%, the maximum tensile strength and microhardness of the composite solder alloy are 68.9 MPa and 23.4 HV, respectively, representing a 37% increase in tensile strength and a 24% increase in microhardness compared to the base solder alloy. However, the elongation decreases from 44.2% for the base solder alloy to 40.6%. When the CeO_2_ addition amount increases to 1.5 wt%, the tensile strength of the composite solder alloy decreases to 61.6 MPa, and the corresponding elongation decreases to 30.6%.

**Figure 7:**
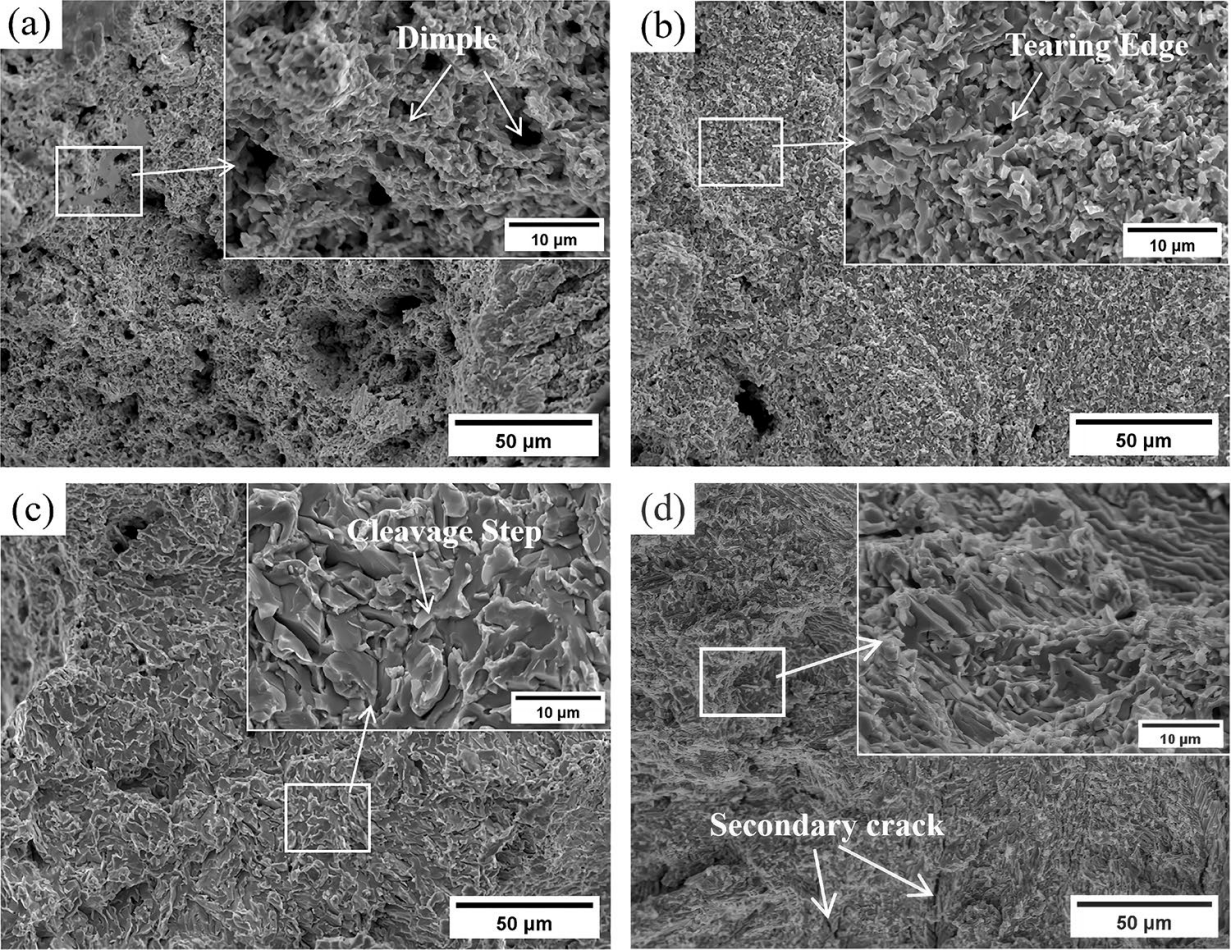
SEM image of tensile fracture morphology of Sn58Bi-*x*CeO_2_(x = 0 ~ 1.5 wt%) composite solder (**a**) 0 wt%; (**b**) 0.5 wt%; (**c**) 1.0 wt%; (**d**) 1.5 wt%.

The tensile fracture morphology of the Sn58Bi-*x*CeO_2_ composite solder alloy is shown in Fig. 7. From Fig. 7, it can be observed that the tensile fracture of the Sn58Bi solder exhibits a ductile fracture characterized by dimples and tearing edges (Fig. 7a). When the addition amount of CeO_2_ nanoparticles is 0.5 wt%, the microstructure of the composite solder alloy is significantly refined, and the tensile fracture is mainly composed of fine dimples and tearing edges (Fig. 7b). The fracture mode remains ductile. With the addition amount of CeO_2_ nanoparticles exceeding 0.5 wt%, the primary β-Sn phase in the composite solder coarsens, and the secondary Bi particles increase. Both Bi particles and CeO_2_ nanoparticles play a role in dispersion strengthening^21^. Under their combined action, the tensile strength and microhardness of the solder increase, while the elongation decreases. More cleavage planes and secondary cracks appear on the tensile fracture surface (Fig. 7c), and the fracture mode shifts to a brittle-ductile mixed fracture, with brittle fracture being dominant.

Excessive addition of CeO_2_ nanoparticles at a concentration of 1.5 wt% manifests distinct characteristics in the composite solder (Fig. 7d). Notably, the fracture surface reveals an abundance of cleavage planes, arranged in a fishbone-like pattern, alongside noticeable voids. When subjected to tensile stress, secondary cracks propagate along grain boundaries, while the accumulation of voids exacerbates fracture, consequently diminishing the material's tensile strength.

Hence, to achieve better comprehensive performance in the Sn58Bi-*x*CeO_2_ composite solder, the addition amount of CeO_2_ nanoparticles should not exceed 0.5 wt%.

### Interfacial microstructure of solder joint

**Figure 8:**
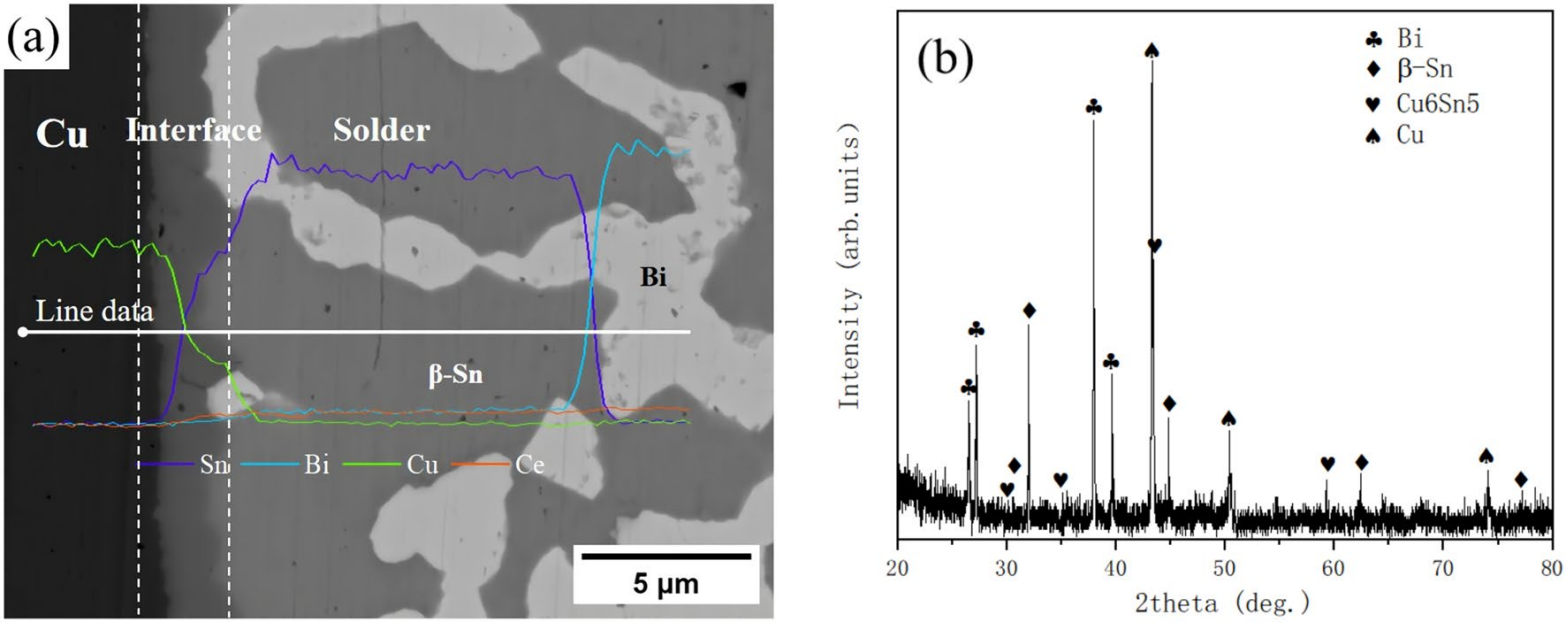
Micromorphology, EDS analysis, and XRD diffraction pattern of Sn58Bi-0.5CeO_2_/Cu solder joint (**a**) micromorphology and EDS analysis; (**b**) XRD diffraction pattern.

The microstructure, line scan, and XRD diffraction pattern of Sn58Bi-0.5CeO_2_/Cu solder joint are illustrated in Fig. 8. As depicted in Fig. 8a, the Sn58Bi-0.5CeO_2_/Cu solder joint can be divided into three regions: the copper substrte, the interface, and the solder seam. Based on the line scan and XRD diffraction analysis (Fig. 8b), it is evident that the solder seam is located in the right region, primarily consisting of a layered eutectic structure, primary β-Sn phase, and secondary Bi particles. The black region on the left side represents the copper substrate. Between the copper substrate and the solder seam, there exists an interface region containing an IMC (Cu_6_Sn_5_ + Cu_3_Sn) layer. Due to the relatively low soldering temperature and short soldering time, the Cu_3_Sn layer is thin and not detected in the experiment^22^. Some Bi phases are attached to the upper part of the interface IMC layer, formed due to the increase in Bi atomic concentration after the reaction between Sn and the Cu substrate, resulting in precipitation from the β-Sn phase.

### Microstructure evolution of solder joints during electromigration

**Figure 9:**
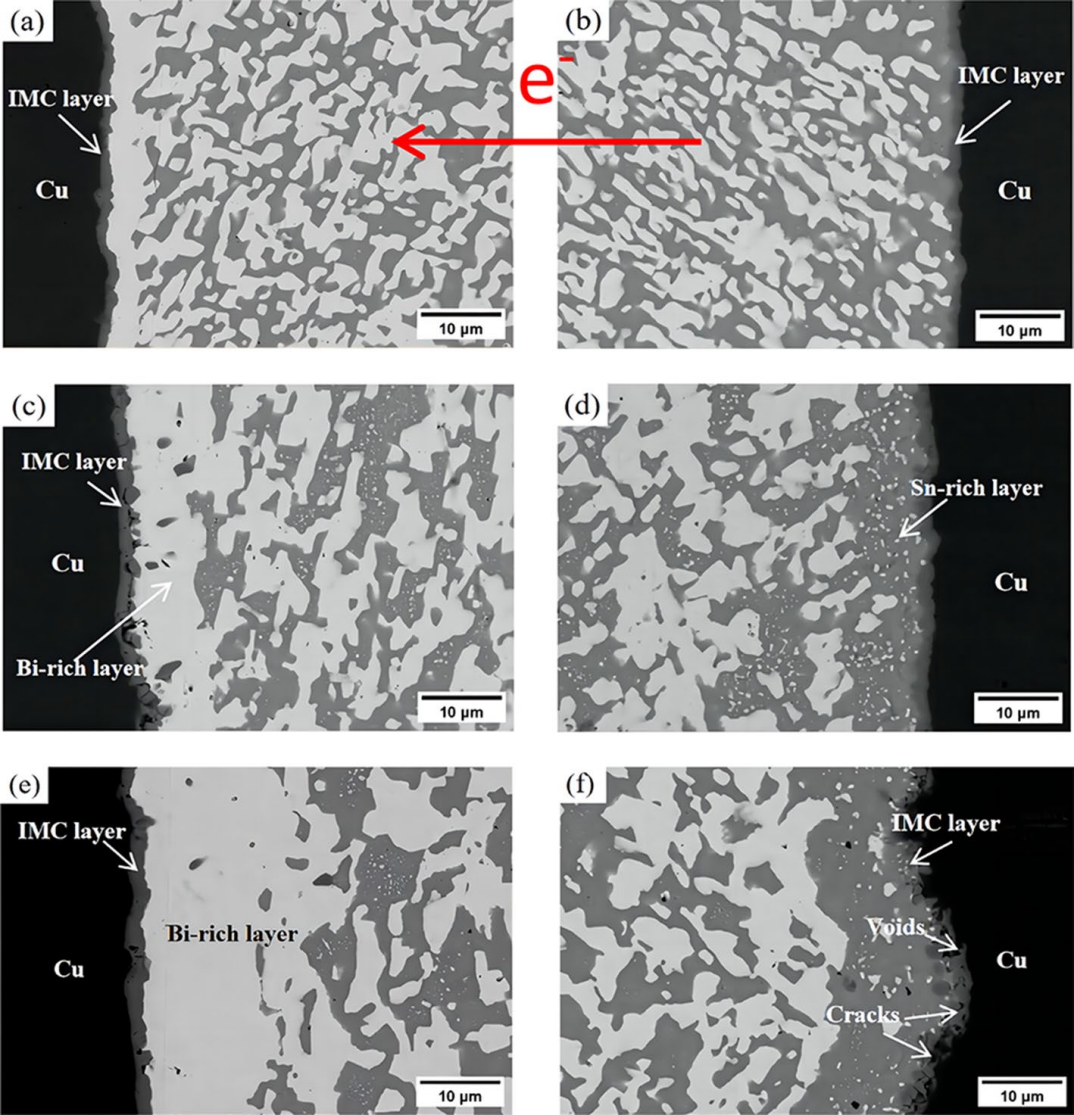
The microstructure of Sn58Bi/Cu solder joints at different current stressing times: (**a**) anode (96 h); (**b**) cathode (96 h); (**c**) anode (288 h); (**d**) cathode (288 h); (**e**) anode (480 h); (**f**) cathode (480 h).

**Figure 10:**
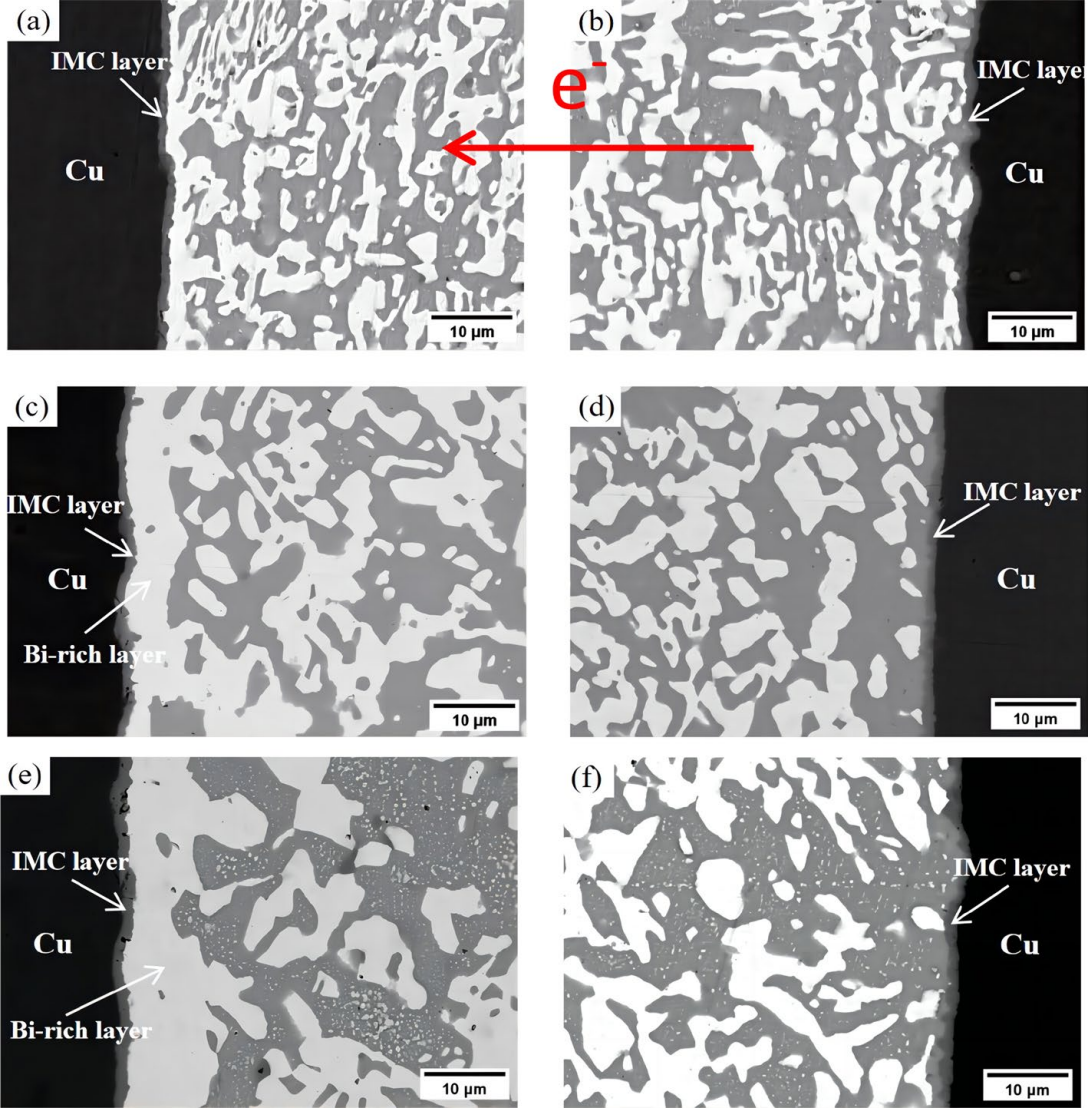
The microstructure of Sn58Bi-0.5CeO_2_/Cu solder joints at different current stressing times: (**a**) anode(96 h); (**b**) cathode (96 h); (**c**) anode (288 h); (**d**) cathode (288 h); (**e**) anode (480 h); (**f**) cathode (480 h).

The microstructure of Sn58Bi/Cu and Sn58Bi-0.5CeO_2_/Cu solder joints at different current stressing times are illustrated in Figs. 9 and 10, respectively. It can be observed that as the current stressing time increases, a continuous and dense Bi-rich layer forms at the anode in the Sn58Bi/Cu solder joint (Fig. 9a,c,e). The Bi phase at the cathode gradually refines from a blocky to a fine granular morphology (Fig. 9b,d,f). In comparison to the Sn58Bi/Cu joint, the Sn58Bi-0.5CeO_2_/Cu joint showcases a thinner anodic Bi-rich layer (Fig. 10a,c,e) and a more even distribution of the cathodic Bi phase (Fig. 10b,d,f).

When the current stressing time reaches 480 h, the eutectic structure in the solder seam coarsens, and the Bi phase changes from a fibrous to a blocky morphology. The cathodic Bi phase gradually disappears, forming a Sn-rich layer (Fig. 9f). The cathodic interface IMC layer transitions from a fan-shaped structure to a thickened mountain-like morphology (Fig. 9f), with numerous microcracks and micropores appearing at the solder seam/IMC interface.Due to the obstruction by the rich Bi layer, the anodic IMC layer adheres to the rich Bi layer and exhibits a flattened morphology.

In the Sn58Bi-0.5CeO_2_/Cu joint after 480 h of current stress, no Sn-rich layer is observed at the cathode (Fig. 10f). Instead, numerous fine Bi particles are dispersed on the β-Sn phase in the solder seam. The interface IMC layer maintains its fan-shaped structure without significant defects.

**Figure 11:**
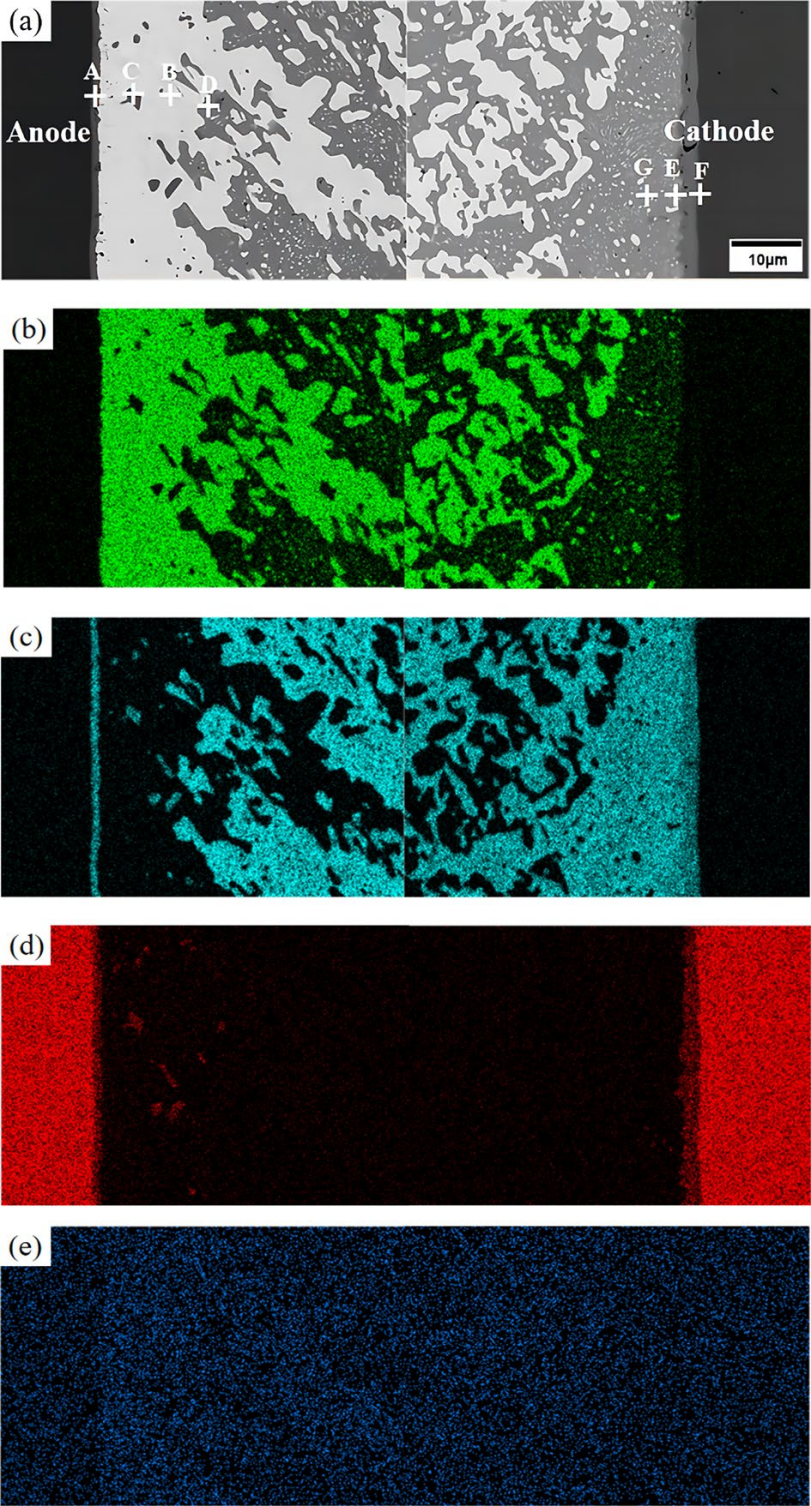
The elemental mapping of Sn58Bi-0.5CeO_2_/Cu solder joint after current stressing for 600 h (**a**) microstructure; (**b**) Bi; (**c**) Sn; (**d**) Cu; (**e**) Ce.

The elemental mapping and selected areas EDS analysis results of the Sn58Bi-0.5CeO_2_/Cu solder joint after 600 h of current stress are shown in Fig. 11 and Table 1, respectively. From Fig. 11, it can be observed that after 600 h of current stress, a distinct Sn-rich layer forms at the cathode in the Sn58Bi-0.5CeO_2_/Cu solder joint. In the Bi-rich layer at the anode, Cu_6_Sn_5_ phases are embedded. Combined with the analysis in Table 1, CeO_2_ is mainly distributed in the Bi-rich layer at the anode and in the IMC layer. This is because during electromigration, Bi atoms migrate towards the anode, and CeO_2_ nanoparticles act as rapid diffusion channels for Bi elements at the interface with the substrate. Consequently, the concentration of Bi atoms increases near the CeO_2_ nanoparticles, leading to the precipitation of the Bi-rich phase in their vicinity.

**Table 1:** EDS analysis of selected areas of solder joint.

Elements (at.%)	Point A	Point B	Point C	Point D	Point E	Point F	Point G
Sn	58.9	0.9	57.6	95.7	64.3	35.1	98.7
Bi	6.7	98.0	8.9	3.0	–	6.5	1.3
Cu	33.6	–	33.5	0.9	34.7	58.4	–
Ce	0.8	1.1	–	0.4	1.0	–	–

**Figure 12:**
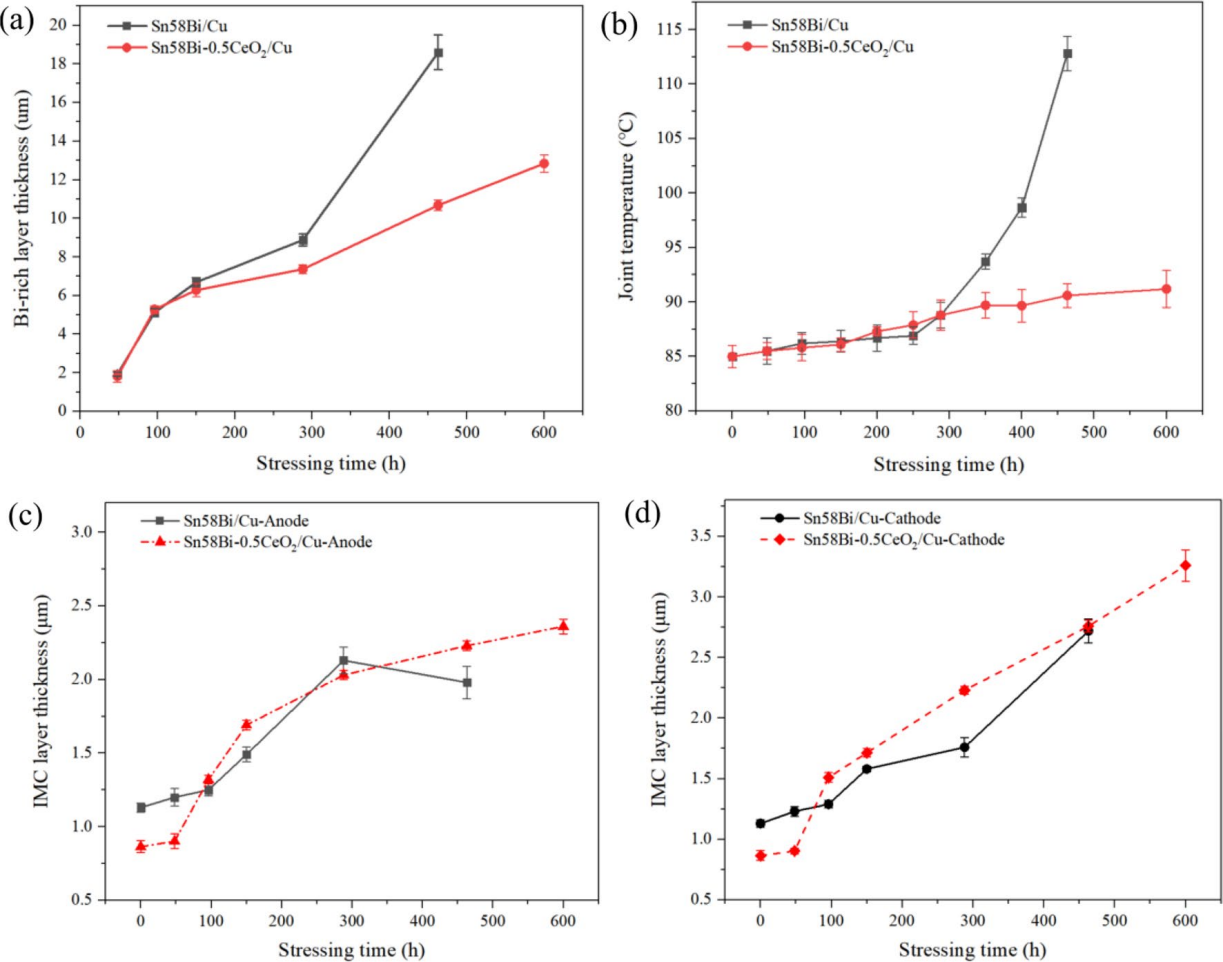
The variations in temperature, interface IMC and Bi layer thickness during the current stressing process of solder joints (**a**) Bi-rich layer thickness; (**b**) solder joint temperature; (**c**) anodic IMC layer thickness; (**d**) cathodic IMC layer thickness.

The variations in temperature, interface IMC and Bi layer thickness during the current stressing process of solder joints are shown in Fig. 12. From Fig. 12, it can be observed that the addition of CeO_2_ nanoparticles suppresses the growth of the anodic Bi-rich layer during electromigration. From 0 to 288 h of current stress, there's a gradual increase in the thickness of both the anodic Bi-rich layer (Fig. 12a) and the IMC layer (Fig. 12c,d), as well as the temperature of the joint (Fig. 12b), exhibit a consistent rise. However, after surpassing 288 h of current stress, the temperature of the Sn58Bi/Cu solder joint rapidly increases. Additionally, the thickness of the anodic IMC layer thickness decreases (Fig. 12c), while the growth rate of the cathodic IMC layer thickness accelerates (Fig. 12d). Under 480 h of electrification, the average thickness of the anodic Bi-rich layer in the Sn58Bi-0.5CeO_2_/Cu solder joint is 10.7 µm, which is 42.5% lower than that of the Sn58Bi/Cu joints.

During the electromigration interface reaction in Sn58Bi-*x*CeO_2_/Cu solder joints, it is necessary to consider the diffusion flux ${J}_{chem}$ caused by the concentration gradient of Cu, Sn, and Bi atoms, as well as the diffusion flux ${J}_{EM}$ driven by the electron wind force^23,24^. Therefore, the atomic diffusion flux J during the electromigration process is given by^25^:

1 $$J={J}_{chem}+{J}_{EM}=D\frac{dC}{dx}+C\frac{D}{KT}{Z}^{*}e\rho j$$

**Figure 13:**
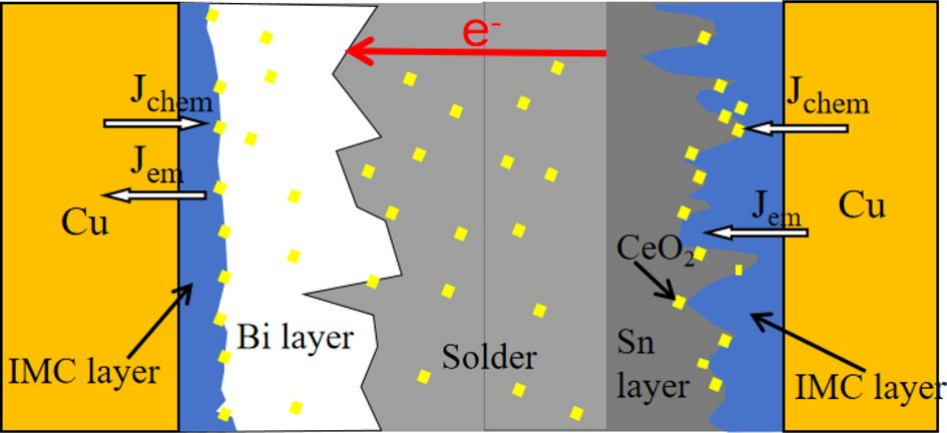
Schematic diagram of Cu atomic flux at SnBi-*x*CeO_2_/Cu interface under current stress^27^.

From Eq. (1), it is evident that the electromigration behavior involves the accumulation over time under the combined influence of thermal energy and electron wind force. The migration of Cu atoms under current stressing is illustrated in Fig. 13. From the Fig. 13, it can be observed that the direction of the ${J}_{chem}$ of Cu atoms at the anode is opposite to that of the ${J}_{EM}$, and ${J}_{chem}$<${J}_{EM}$, indicating that the diffusion of Cu atoms at the anode is mainly driven by the concentration gradient, moving from the Cu substrate to the IMC layer. When the current is applied for 0 ~ 288 h, the anodic IMC layer shows a growing trend. At the cathode, where the direction of ${J}_{chem}$ is the same as ${J}_{EM}$, it manifests as a faster growth rate of the cathodic IMC layer compared to the anode. In the initial stage of electromigration, Bi phases are dispersed in the solder seam region, where the diffusion of Bi atoms is less influenced by the concentration gradient and primarily driven by the electron wind force^26^. Under the influence of the electron wind force, Bi atoms continuously migrate towards the anode, leading to the formation of a Bi-rich layer.

From 288 to 480 h of current stress, a Bi-rich layer with a thickness ranging from 6 to 20 µm forms at the anode of the solder joint. Due to the significant difference in electrical conductivity between Bi (0.77 S/μm) and Sn (9.1 S/μm), the accumulation of the Bi-rich layer and the presence of cracks and voids at the cathode cause an increase in the electrical resistance of the solder joint. This leads to an increase in Joule heating, causing the temperature of the Sn58Bi/Cu solder joint to rise sharply. After 480 h of current stress, the temperature of the Sn58Bi/Cu joint increases to 112.5 ± 3 °C, corresponding to a T/T_M_ value of 0.82 relative to the melting point of the solder (138 °C). Intensified diffusion between the various elements occurs, predominantly via bulk diffusion^28^.

Near the cathodic IMC region, the Bi phase is decomposed, forming a rich Sn layer. The hindrance effect of the Bi phase on the growth of IMC is reduced, resulting in an accelerated growth rate of the cathodic IMC layer, and eventually the IMC shape is transformed into a thickened mountain-like morphology.

Due to the hindrance from the Bi-rich layer, the concentration of Sn atoms decreases at the anodic IMC, resulting in a reduction in the reaction rate between Cu and Sn, where the growth rate of Cu_6_Sn_5_ is lower than its consumption rate. This manifests as a thinning of the anodic IMC layer.

Under the influence of the electron wind force-driven vacancy-atom exchange mechanism^29^, vacancies migrate towards the cathode. This results in the gradual increase in microcracks at the interface between the IMC and the soldering seam, which merge to form voids. The increase in defects at the interface leads to an increase in the electrical resistance of the joint, causing a further rise in joint temperature and exacerbating the diffusion process.

### Shear force changes in solder joints after electromigration

**Figure 14:**
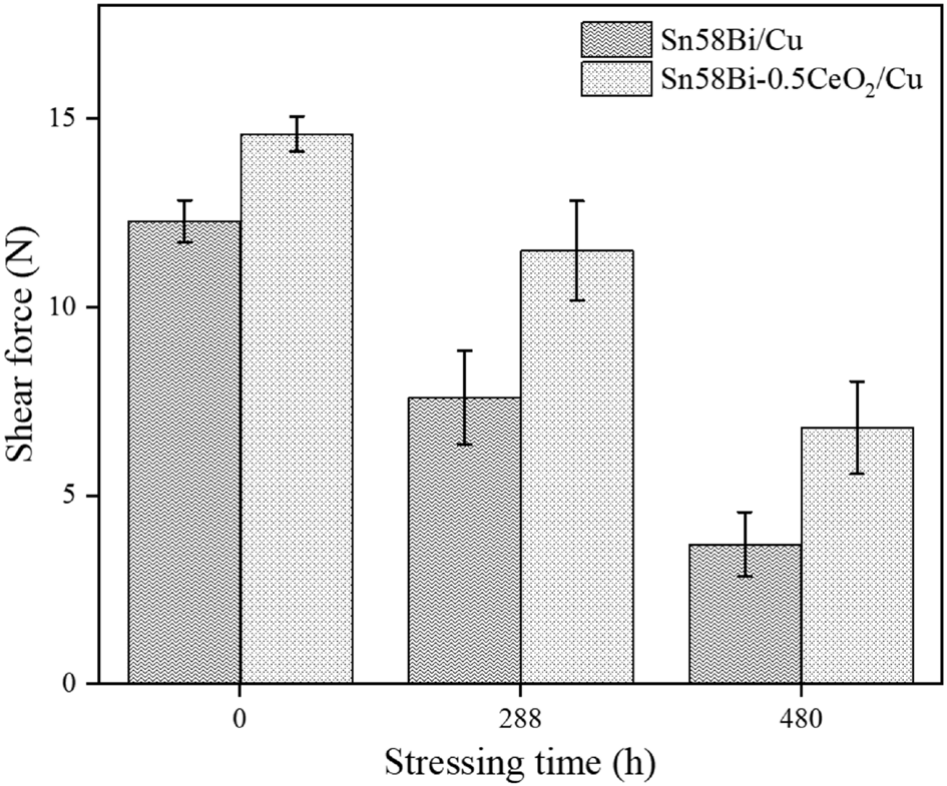
The shear force of solder joints under different current stressing time.

The effect of current stressing time on the shear force of solder joints is depicted in Fig. 14. As shown in the Fig. 14, it can be observed that when no current is applied, the average shear force of the Sn58Bi-0.5CeO_2_/Cu solder joint is 14.6 N, which is 18.7% higher than that of the Sn58Bi/Cu solder joint. At current stressing times of 288 h and 480 h, the average shear force of the Sn58Bi-0.5CeO_2_/Cu solder joint decreases by 21.2% and 53.4%, respectively, while that of the Sn58Bi/Cu solder joint decreases by 38.2% and 69.9%, respectively. This indicates that adding CeO_2_ nanoparticles not only enhances the mechanical properties of the Sn58Bi/Cu solder joint but also mitigates to some extent the reduction in mechanical properties caused by electromigration damage during current stressing.

**Figure 15:**
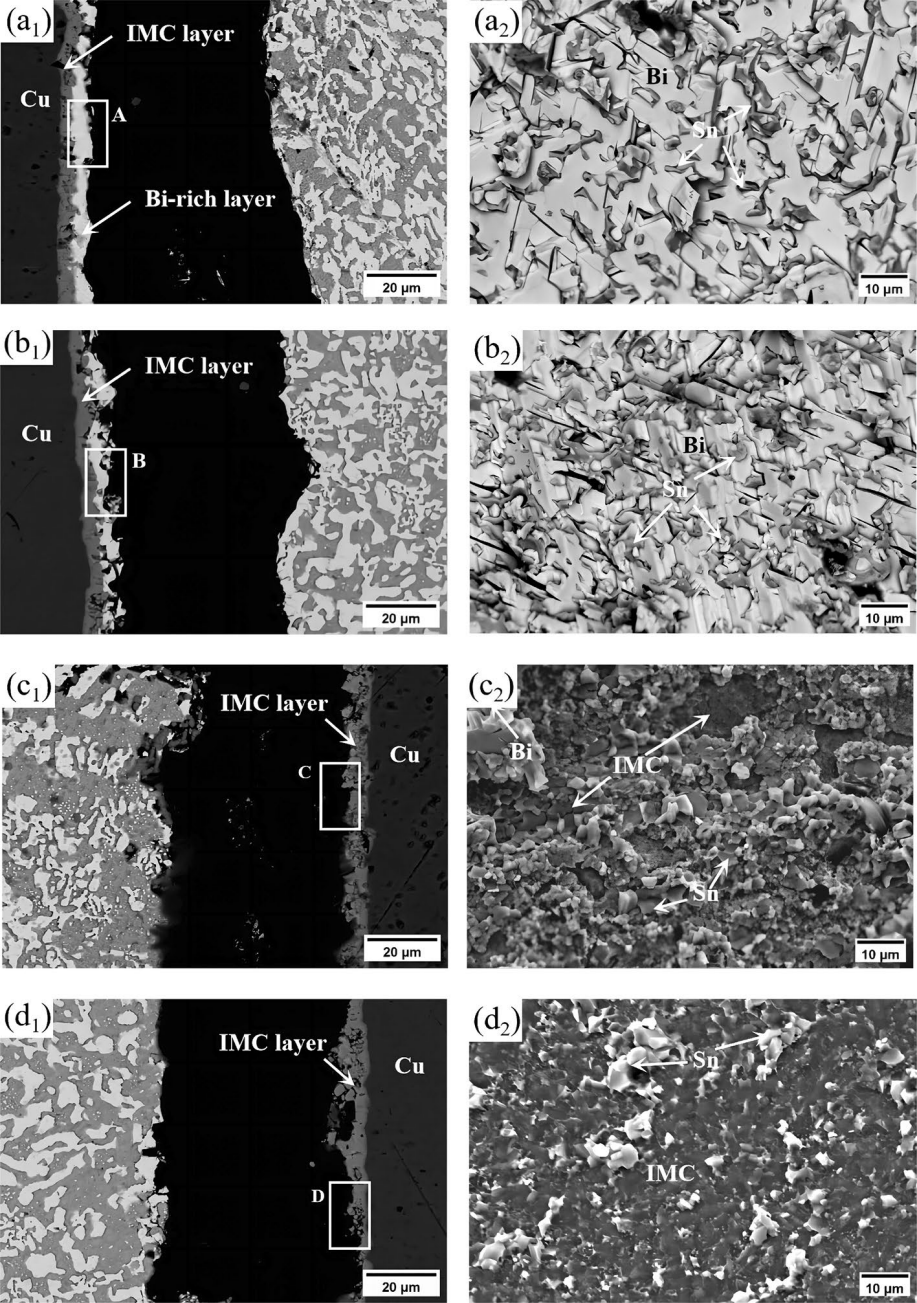
Shear fracture morphology of solder joints at different current stressing time: (**a**_**1**_) Sn58Bi/Cu (288 h); (**a**_**2**_) Top view of micro area A; (**b**_**1**_) Sn58Bi-0.5CeO_2_/Cu (288 h); (**b**_**2**_) Top view of micro area B; (**c**_**1**_) Sn58Bi/Cu (480 h); (**c**_**2**_) Top view of micro area C; (**d**_**1**_) Sn58Bi-0.5CeO_2_/Cu (480 h); (**d**_**2**_) Top view of micro area D.

The morphologies of shear fracture surfaces and their selected areas spectrum element analyses under different current stressing times are shown in Fig. 15 and Table 2, respectively. From Fig. 15, it can be seen that at a current stressing time of 288 h, shear fracture occurred at the anode (Fig. 15a,b).The shear fracture surface of Sn58Bi/Cu solder joints exhibits a brittle fracture composed of cleavage planes of Bi phase arranged in layered structure and secondary cracks (Fig. 15a). After adding 0.5 wt% CeO_2_ nanoparticles, the cleavage plane size of the shear fracture reduced, and the brittleness decreased. Combined with Table 2, it can be inferred that the fracture of the Sn58Bi/Cu solder joint mainly occurred at the anodic Bi rich layer, and with the addition of CeO_2_ nanoparticles, the fracture position shifted to the anodic Bi-rich/IMC interface.

**Table 2:** EDS analysis of the shear fracture selected area of the solder joint in Fig. 15 (At.%).

Area	Sn	Bi	Cu	Ce
A	4.4	95.6	–	–
B	8.1	90.3	1.5	0.1
C	61.1	5.7	33.2	–
D	75.5	2.4	21.9	0.2

At a current stressing time of 480 h, shear fracture occurred at the cathode (Fig. 15c,d).The shear fracture surface of Sn58Bi/Cu exhibits a brittle fracture characterized by flat cleavage planes composed of IMC , while Sn58Bi-0.5CeO_2_/Cu shows a mixed brittle-ductile fracture, mainly composed of fine dimples of β-Sn and cleavage planes of IMC. (Fig. 15c,d). Combined with Table 2, it can be inferred that the shear fracture position of the Sn58Bi/Cu solder joint was in the cathodic IMC layer, and with the addition of CeO_2_ nanoparticles, the fracture position shifted to the Sn-rich/IMC interface. The addition of CeO_2_ nanoparticles reduced the progression of electromigration in the solder joint, mitigated the formation of cathodic IMC defects, and improved the service life of the solder joints.

## Conclusion

- The addition of CeO_2_ nanoparticles has little effect on the melting point and electrical conductivity of Sn58Bi solder. The addition of CeO_2_ nanoparticles at 0.125 ~ 0.5 wt% refines the eutectic structure of Sn58Bi solder. The maximum tensile strength of the composite solder is achieved at 68.9 MPa with an addition of 0.5 wt%, which is a 37% improvement over the base solder. The tensile fracture mechanism exhibits a ductile fracture mode characterized by fine dimples and tearing edges.
- CeO_2_ nanoparticle-reinforced Sn58Bi solder can achieve excellent solderbility with Cu substrates. With increasing current stressing time, noticeable electromigration phenomena occur in the solder joints, resulting in the formation of rich Bi and Sn layers at the anode and cathode, respectively. The IMC layer exhibits asymmetric growth, transitioning from a fan-shaped morphology to a flattened structure at the anode and to a thickened mountain-like morphology at the cathode.
- Adding CeO_2_ nanoparticles improves the mechanical properties of Sn58Bi/Cu solder joints. It also helps to mitigate the decrease in mechanical performance caused by electromigration damage during current application to some extent. The Sn58Bi-0.5CeO_2_/Cu solder joints effectively suppresses the growth of the anodic Bi-rich layer. Over the current stressing period of 288 ~ 480 h, the fracture position shifts from the anodic IMC/Bi-rich interface to the cathodic Sn-rich/IMC interface. The fracture mechanism transitions from a brittle fracture characterized by plate-like cleavage to a ductile–brittle mixed fracture with fine dimples and cleavage.

## Data Availability

The data used to support the findings of this study are included within the article.
